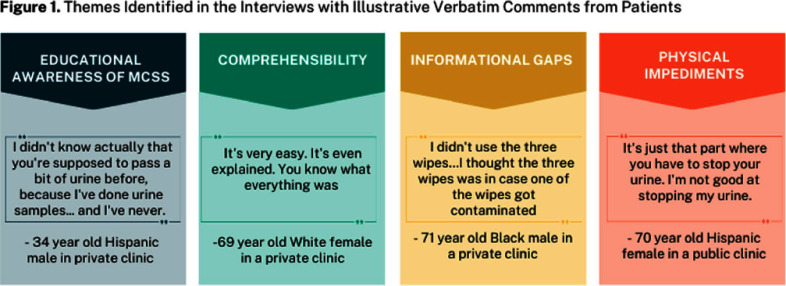# Patient Perceptions of an Educational Intervention to Reduce Urine Sample Contamination

**DOI:** 10.1017/ash.2025.391

**Published:** 2025-09-24

**Authors:** Ashley Collazo, Barbara Trautner, Rosalyn Campbell, Azalia Mancera, Kiara Olmeda, Fabrizia Faustinella, Mohamad Sidani, Lisa Danek, Kenneth Barning, Michael Paasche-Orlow, Roger Zoorob, Larissa Grigoryan

**Affiliations:** 1 Baylor College of Medicine; 2 Baylor College of Medicine; 3 Baylor College of Medicine; 4 Baylor College of Medicine; 5 Baylor College of Medicine; 6 Baylor College of Medicine; 7 Baylor College of Medicine; 8 Baylor College of Medicine; 9 Baylor College of Medicine

## Abstract

**Background:** Urine cultures are the gold standard for urinary tract infection (UTI) diagnosis and are becoming increasingly important to guide antibiotic choice. However, when samples are not collected properly they can become contaminated. In our primary care safety-net clinics, 694 (55%) of all urine samples collected from non-catheterized patients were contaminated, which led to one in five patients receiving unnecessary antibiotics and a waste of lab resources. We have developed a bilingual (English and Spanish), multicultural educational intervention that includes an animated instructional video and a flyer with pictorial instructions providing step-by-step guidance for collecting a midstream clean catch (MSCC) sample. **Methods:** A patient advisory board (PAB) was assembled to review our materials, recruiting patient representatives from 2 private and 2 public primary care clinics in Harris County. The PAB included 7 Hispanic patients (2 female), 3 Black patients (2 female), and 2 White patients (1 female), of which half were bilingual (50%). Each board member received a urine sample collection kit plus our educational flyer. One-on-one interviews were conducted with each PAB member, during which the educational video and flyer were shown. Using thematic analysis, the interview data was condensed into themes. (Figure 1) **Results:** There was a lack of awareness of how to collect a MSCC among patients. Most found the educational material to be helpful in clarifying the process, and the graphical elements were especially appreciated by those with difficulty reading. Patients reported confusion around appropriate use of the wipes and lack of pictorial directions for handwashing. Physical impediments reported by patients with the MCSS process included difficulty balancing and stopping urine flow for a mid-stream sample, particularly for elderly females with medical comorbidities. **Conclusions:** Our educational intervention was well received by a sociodemographically diverse group of patient representatives with minimal improvements needed. Future work involves implementation of this educational intervention into primary care workflows and evaluating its effectiveness in reducing urine contamination. **Acknowledgments:** This work is funded by R01HS029489-02 from the Agency for Healthcare Research and Quality (AHRQ). Dr. Collazo is funded by the Department of Health and Human Services, Health Resources and Services Administration (T32HP10031).

**Figure:**